# Caregiver-Reported Barriers To Mental Health Service Use for Asian American Youth

**DOI:** 10.1007/s10903-025-01810-x

**Published:** 2025-11-01

**Authors:** Amy Hyoeun Lee, Lillian Alford, Nicole Lui

**Affiliations:** Hofstra University, Hempstead, United States

**Keywords:** Asian american youth, Mental health disparities, Service utilization, Barriers, Cultural factors

## Abstract

Asian American (AsAm) youth experience persistent disparities in mental health service use, but quantitative data are lacking in existing investigations of barriers in this population. Here, we sought to characterize key barriers to youth mental health services across multiple domains (i.e., structural, perceptual, cultural, trauma-specific) and explore associations between barriers, youth symptoms, and service use. AsAm caregivers (*N* = 149, *M*_age_ = 39.23, 55% female) completed the study online, reporting on barriers, youth psychopathology, and past-year service use. Caregivers reported significant barriers to seeking youth mental health services in each barrier domain. For youth mental health needs, caregivers reported using medical services most frequently, followed by school-based care and therapy/counseling. Caregivers of youth with clinically significant symptoms (clinical subgroup; *n* = 51) endorsed barriers more frequently compared to caregivers of youth without (*n* = 98), and unique key barriers emerged in the clinical subgroup. In the overall sample, mean barrier scores were positively correlated with perceived need for therapy and school-based support services, but not with medical support services. Barrier scores were positively correlated with past-year use of school-based support services, but not with therapy or medical services. Finally, barriers were significantly correlated with youth mental health symptoms, suggesting that caregivers of youth with more severe symptoms perceived more barriers to care. Addressing culturally relevant barriers, in addition to universal barriers, could improve access and engagement with mental health services for AsAm youth. Further research is needed to replicate and generalize these findings to broader samples of AsAm youth and caregivers.

## Background

Asian Americans represent the fastest growing racial group in the U.S. and are projected to become the largest immigrant group by 2055 [1]. Among U.S. communities of color, they are also the *least likely* to use mental health services, with these disparities extending to Asian American (AsAm) youth [2]. Despite this, specific factors affecting access to care for children’s mental health needs in this population are understudied. Asian Americans are notably underrepresented in research, with less attention given to health disparities in this population relative to other racial groups [3]. Given the low rates of mental health service use among Asian Americans and the lack of established research findings explaining such disparities, identifying barriers faced by AsAm youth in accessing mental health services is an important first step in addressing the longstanding mental health treatment gap [4] for AsAm communities.

Access to youth healthcare services, including mental health care, is typically facilitated by their caregivers [5]. As such, it is important to understand barriers to mental health care for AsAm youth from caregivers’ perspectives. Much of the prior research on caregivers’ help-seeking behaviors pertaining to their child’s mental health has not included Asian Americans [3]. Moreover, a recent systematic review synthesizing barriers and facilitators of mental health services for Asian American youth identified 6 studies using qualitative approaches, but no quantitative studies [6]. Thus, more research is needed to better understand the ways in which Asian Americans’ cultural values and beliefs impact the identification of youth mental health concerns, their perceived need for services, and their subsequent help-seeking behaviors [7]. The aim of this quantitative study was to describe caregiver-reported barriers to mental health services and patterns of service use for AsAm youth and to explore associations between barriers, youth symptoms, and service use.

### Barriers To Mental Health Services

Gopalan et al. [8] identified barriers to child mental health treatment engagement among families in (1) structural, (2) perceptual/attitudinal, and (3) cultural domains. In the present study, we additionally considered the role of (4) trauma-specific barriers given the relevance of historical, immigration, and interpersonal trauma for mental health outcomes among racial ethnic minority youth in the U.S [9]. Below, we briefly review the literature in each of these domains as they pertain to AsAm youth and caregivers.

**Structural.** Structural barriers encompass systematic or logistical factors that impact one’s ability to obtain treatment, including accessibility, affordability, and convenience of the treatment options available. Known structural barriers affecting AsAm communities include financial barriers [10, 11], provider shortage [10], and the cost-benefit of help-seeking [12].

***Financial Barriers.*** Racial and ethnic communities face disparities in access to health insurance coverage. The implementation of the Affordable Care Act (ACA) resulted in a significant decrease in uninsurance rate among Asian Americans between 2017 and 2018, but improvements varied among different Asian subgroups. For instance, Korean and Vietnamese Americans were significantly more likely to be uninsured compared to their Indian, Chinese, and Filipino American counterparts [13]. While health insurance coverage has been improved through ACA’s coverage expansions, challenges with healthcare insurance remain for this population. For instance, Asian Americans own half of minority-owned businesses and faced the risk of losing both their businesses and healthcare coverage due to the pandemic-driven recession [13]. Moreover, levels of acculturation including English language proficiency affect employment status, which could in turn impede the ability to seek and receive mental health services [11]. Financial barriers also apply to out-of-pocket costs of treatment services, including co-pays, deductibles, and up-front fees in private pay clinics. Taken together, insurance coverage and financial means are instrumental for ensuring timely access to treatment, and significant financial barriers persist among Asian Americans [14]. Given the growth of AsAm populations in the U.S., there is a need for efforts to address these disparities, which may include comprehensive healthcare reform, dissemination of evidence-based mental health services in community-based settings, and targeted augmented interventions to improve access and engagement for this population [11].

***Provider Shortage.*** There is an estimated ratio of 1 mental health provider (e.g., masters-level therapists, psychologists, psychiatrists) for every 350 individuals in the U.S [15]. Mental health resources are not evenly distributed, leading to even more provider shortages in densely populated areas [15]. For the AsAm community, an additional systemic barrier includes the lack of culturally responsive and AsAm providers. Research shows that culturally responsive care and providers who match the racial and ethnic identity of their clients tend to result in higher engagement and lower rates of attrition [16]. For instance, Meyer and Zane [17] found that clients from ethnic minority groups considered issues related to race and ethnicity as more important in their mental health treatment compared to white clients. They also demonstrated that cultural factors were significantly associated with treatment accessibility, satisfaction, and perceptions of treatment quality for ethnic minority clients but not white clients [17]. For Asian Americans, there are notable cultural differences in approaches to emotions, distress, and healing [18]. These differences are then magnified with the significant underrepresentation of AsAm providers. In 2019, the percentage of AsAm psychologists within the mental health workforce was 30% less than the proportion of Asian Americans in the general population [19]. This underrepresentation of AsAm providers poses as a substantial structural barrier given that clients perceive clinicians from the same communities as them as more trustworthy [19].

***Cost-Benefit of Help Seeking.*** AsAm youth and families may be reluctant to seek care because treatment is perceived as less necessary or helpful. Experiences of ongoing oppression or racial discrimination could increase general distrust of systems built for and by majority-dominant institutions [12]. Furthermore, factors such as ongoing discrimination, acculturation, historical trauma, and related sociocultural issues that impact many people of color may not be routinely addressed nor discussed in usual care settings due to being perceived as difficult to address or inappropriate [20]. When families do not perceive mental health treatment settings as safe spaces to target problems that affect their daily functioning, they could conclude a lack of need for services and not seek treatment even if they have the means to do so. This may in turn reinforce a preference for informal sources of support observed among Asian Americans [21].

**Cultural & Attitudinal.** The AsAm community encompasses over 40 ethnicities and 100 languages. Compared to those who are fluent in English, Asian Americans with diverse language needs are more likely to underutilize healthcare services and receive lower quality care [22]. Language barriers have been further intensified with the rapid shift to telehealth services during the COVID-19 pandemic, given that many telehealth systems were not prepared to accommodate diverse language needs. As such, researchers have highlighted the need for healthcare systems to provide in-language information and interpretation services to address disparities in youth mental health services [23]. Language barriers have implications for AsAm youth in need of care, especially for youth from immigrant families whose caregivers have limited English language proficiency.

In many Asian cultures, the family is seen as a hierarchical and collective unit. Thus, striving for family wellbeing remains a priority, and behaviors that might bring dishonor to the family are discouraged [24]. Given this, seeking support for individual mental health concerns may be seen as bringing shame to the family and something to be handled by the individual or within the family. Compared to majority group members, stigma against mental illness is higher among racial and ethnic minorities [24, 25]. In many AsAm cultures, the effects of stigma extends beyond the individual. “Saving face” is a cultural value related to their collective orientation in many Asian communities and describes the need to preserve a respectable public image for the sake of community propriety [7]. AsAm youth who internalize this value may suppress their mental health needs to avoid community rejection and protect the family image. This intergenerational stigma could deter youth from confiding in caregivers and seeking support when mental health needs arise [7].

Due to differing cultural norms around emotional expression, disclosures of personal or social problems may be viewed as unacceptable in AsAm communities [24]. Yet, current mental health treatments emphasize verbal and behavioral expressions of emotions. For many Asian Americans, such vulnerable admissions may be viewed as reflecting poorly on themselves and their families. Thus, the perceived negative consequences of emotional expression or disclosure may further hinder help-seeking. Among people of color, concerns about involuntary hospitalization may also be a concern, given a history of negative interactions with law enforcement and distrust of the medical system [24]. These individuals may also believe that such agencies will be further involved in hospitalization procedures and treat them poorly [10].

Among racial ethnic minorities, discrimination is also a known risk factor for poor mental health outcomes. For AsAm youth, racial discrimination is correlated with poor self-esteem, depressive symptoms, and the internalization of racialized stereotypes [18]. The “Model Minority Myth,” defined as the false view of Asian Americans as having achieved “successful assimilation into a white dominant society and as living examples of advanced [achievement], in spite of the persistent color line and because of their racial differences” [26], may be a barrier uniquely affecting AsAm youth. To uphold the stereotype, many AsAm youth experience undue pressure to excel academically and achieve occupational success. Internalization of this stereotype also perpetuates self-stigma of experiencing distress and receiving care [24]. As such, psychological and behavioral problems exhibited by Asian Americans are also overlooked by youth themselves and others, limiting the opportunity for timely linkage to care [27].

**Trauma-Specific.** Trauma-specific barriers may also be crucial to better understanding the low rates of mental health service use within AsAm communities. Trauma-related barriers stem from a variety of interrelated sources including historical, immigration, racial, and pandemic-related events. These experiences have perpetuated experiences of marginalization, distrust, and intergenerational trauma within the AsAm community [28]. Avoidance is a hallmark symptom of posttraumatic mental health sequelae, and the interaction between cultural values of interdependence, combined with avoidance of trauma-focused discussions, may be especially salient among Asian Americans.

Although few studies have examined trauma exposure among Asian Americans, some research has shown that AsAm youth appear to self-report lower rates of exposure to traumatic events compared to youth from other racial and ethnic minority groups [28]. However, it must be noted that underreporting of such experiences and minimizing their effects may be common in AsAm youth because of cultural norms and values. It is also important to note that some AsAm subgroups (e.g., Chinese, Japanese, Korean) report lower violence exposure and victimization compared to other AsAm ethnic subgroups (e.g., Southeast Asians and Samoans) with greater socioeconomic and demographic disadvantage, potentially masking high rates of trauma exposure when data are aggregated. For instance, in a study conducted with 80 Southeast Asian adolescents, 77.5% had witnessed a violent crime or act of serious physical aggression, while 43.7% had been a direct victim of a crime [29]. While these rates of victimization were higher compared to their white, Hispanic, and African American counterparts, rarely are these experiences identified and addressed [29].

The recent COVID-19 pandemic has also led to increases in racism, microaggressions, and hate crimes toward the AsAm community. From 2019 to 2020, there was a 145% increase in anti-Asian hate crimes across the 16 largest cities in the U.S [30]. These incidents exacerbated the effects of existing racial trauma and were linked to PTSD-related symptoms, such as hypervigilance, flashbacks, avoidance, and somatization [31]. Adding to the history of racial issues that Asian Americans have faced in the U.S., the pandemic exposed layers of trauma of heightened xenophobia, scapegoating, and anti-Asian hate incidents [24]. Given the increase in such incidents intensified by the pandemic, there is a pressing need to better understand trauma-specific barriers to care for AsAm youth, with the aim of improving mental health service utilization.

### The Current Study

Taken together, it is likely that AsAm caregivers who serve as gatekeepers of youth mental health services are directly affected by these structural, cultural, attitudinal, and trauma-specific barriers when considering mental health services for their youth. Caregiver perceptions of barriers, in turn, perpetuate the disparities in access to care for AsAm youth. Yet, limited prior research has examined caregiver-reported barriers to mental health services for AsAm youth, with this research relying primarily on qualitative methods and focused on school-based mental health services [6]. School is an important setting in which youth may be linked with or directly access mental health services, but research accounting for additional service delivery environments (e.g., healthcare systems) is also needed. Better understanding the unique set of barriers for AsAm youth in accessing mental health care broadly has important implications for the development of culturally responsive care and for addressing access disparities for youth in AsAm communities. Thus, the aim of the current quantitative study was to characterize key barriers (i.e., those most frequently endorsed) to mental health care for AsAm youth across multiple barrier domains, describe patterns of formal youth mental health service use, and to explore the associations between barriers, youth mental health symptoms, and service use. Given the lack of prior quantitative research findings in this area [6] and the descriptive aims of this study, we did not delineate hypotheses regarding key barriers. Likewise, although prior studies have found positive associations between youth mental health symptom severity and parent-reported barriers [32], there are no prior studies examining these associations for AsAm caregivers specifically, and the same is true of associations between caregiver-reported barriers and youth service use [33]. Thus, these analyses were exploratory in nature.

## Methods

### Participants and Data Collection

Participants included 149 AsAm caregivers ages 24 to 52 (*M*_age_ = 39.23). Approximately half of the participants identified as female (55%) and were born in the U.S. (56%). Most caregivers (73%) possessed Bachelor’s, Master’s, Doctoral, or Professional Degrees. The remaining 27% completed high school, trade school, or associates degrees. Caregivers reported on their youth ages 6–17 (*M*_age_ = 10.00; 49% female), most of whom were born in the U.S. (94%) and had private health insurance (81%). See Table 1 for additional sample characteristics. Participants were recruited through Prolific (www.prolific.com*)*, a recruitment platform shown to yield higher quality data compared to other crowdsourcing methods [32]. We took this approach to recruitment because of the anticipated difficulty in recruiting our sample of AsAm caregivers, who are less likely to participate in health research compared to other racial ethnic groups [34]. To be eligible, participants had to be ages 18 or above, identify as Asian or AsAm, currently reside in the U.S., and be the primary caregiver of at least one child ages 6 to 17.

**Table 1:** Descriptive statistics of sample characteristics (*N* = 149)

Variable	Caregivers	Youth
*Age, in years*		
M(SD)	39.23 (6.10)	10.00 (3.10)
Range	24–52	6–17
*Sex*		
Female	55.0%	49.0%
Male	45.0%	51.0%
*Ethnicity*	*n* = 41 Chinese *n* = 29 Filipino *n* = 21 Korean *n* = 16 Indian *n* = 15 Vietnamese *n* = 9 Pakistani *n* = 8 Japanese *n* = 3 Bangladeshi *n* = 2 Malaysian *n* = 1 Indonesian *n* = 1 Laotian *n* = 2 Thai *n* = 1 Other	*n* = 38 Chinese *n* = 29 Filipino *n* = 18 Korean *n* = 16 Indian *n* = 17 Vietnamese *n* = 9 Other *n* = 7 Japanese *n* = 7 Pakistani *n* = 3 Bangladeshi *n* = 2 Thai *n* = 1 Indonesian *n* = 1 Laotian *n* = 1 Malaysian
*Nativity*		
Born in the U.S.	55.7%	94.0%
Born outside the U.S.	44.3%	6.0%
*Insurance Status*	--	
Private		80.5%
Public/Medicaid		16.8%
None		2.7%
*Marital Status*		--
Married	81.9%	
Divorced	5.4%	
Separated	2.7%	
Never married	10.1%	
*Household Composition*		--
Two-parent	78.5%	
Single parent	9.4%	
Multigenerational/extended family	12.1%	
*Family Annual Income*		--
$150k+	28.9%	
$125k - $149,999	13.4%	
$100k - $124,999	13.4%	
$75k - $99,999	15.4%	
$50k - $74,999	18.1%	
<$30k	6.0%	

We obtained Institutional Review Board approval for the study prior to beginning data collection. Data collection occurred online from June 2023 to July 2024 using a cross-sectional design. Eligible participants completed informed consent online, and upon providing consent, were administered a series of questionnaires lasting approximately 30 min, completed in a single session (Median time for completion = 24:08). Participants were paid $10 on the platform upon completing the study. Participants who reported having more than one child were asked to select the child who would have most benefited from mental health services in the past year when completing the questionnaires about their child.

### Measures

#### Demographic Questionnaire

Caregivers were asked to report demographics information for themselves and their family (i.e., age, sex, gender, race and ethnicity, country of origin, immigration background, languages spoken, marital status, family structure, age of children, employment status, generational status, education, household income), and for their child (i.e., age, sex, gender, ethnicity, country of origin, insurance status, and disability status).

#### Barriers To Mental Health Treatment Questionnaire for Latina/o/x Caregivers of Children and Adolescents (BTQ-LC [33])

We used the BTQ-LQ to assess caregiver-reported barriers to youth service use. The original measure developed by Marques and colleagues (Barriers to Mental Health Treatment Questionnaire [35]) consists of 23 items. Vázquez et al. [33] introduced an additional item reflecting fear of deportation when validating this scale among Latina/o/x caregivers. The BTQ-LQ yields a global score or subscale scores based on three factors: structural barriers, perceptions of mental health problems, and perceptions of mental health services.

For the current study, we modified the BTQ-LC by adding 7 items reflecting trauma-specific barriers and 4 items pertaining to cultural barriers. These items were constructed based on findings from prior qualitative studies. Specifically, Truss et al. [36] identified trauma-specific barriers including questioning the validity of trauma response, negative emotions and beliefs of self, fear of negative response, difficulties being vulnerable, and social isolation. Wang et al. [37] identified cultural barriers among Asian American immigrant parents including fear of judgment and cultural stigma. Thus, the final modified measure included 35 items (See Table 2). Caregivers were asked to indicate the extent to which each barrier influenced their decision to delay or avoid seeking support services for their child in the last year, using a 5-point scale (1 = *not at all* to 5 = *extremely*). A global score was calculated by taking the average of responses across all 35 items. Subscale scores were computed by taking averaging responses across items in each subscale, with higher scores indicating greater endorsement of barriers in that domain. Internal consistency for the BTQ-LC was excellent in the current sample, α = 0.94 for the global score, and α = 0.86 for structural, α = 0.76 for perceptions of mental health problems, α = 0.87 for perceptions of mental health services, α = 0.87 for cultural, and α = 0.78 for trauma-specific subscales.

**Table 2:** Frequencies of endorsements of barrier items in the overall sample and by clinical status

BTQ Domain/Item		Overall Sample (*N* = 149)		Clinical (*n* = 51)		Non-Clinical (*n* = 98)	
***Structural***		*n*	%	*n*	%	*n*	%
1	I was worried about the cost of treatment	**94**	**63.1**	**41**	**80.4**	**53**	**54.1**
2	My health insurance does not cover treatment for my child	79	53.0	**38**	**74.5**	41	41.8
3	I did not know who to see or where to go for treatment	**90**	**60.4**	36	70.6	**54**	**55.1**
4	I could not get to treatment because of problems with transportation	36	24.2	19	37.3	17	17.3
5	There is no time in my schedule for treatment	79	53.0	35	68.6	44	44.9
6	I could not get an appointment	53	35.6	30	58.5	23	23.2
7	I could not choose the person I wanted my child to see for treatment	59	39.6	33	64.7	26	26.5
16	I was not satisfied with the services that were available	50	33.6	29	56.9	21	21.4
22	I could not find a mental health professional of my child’s same race or ethnicity	45	30.2	23	45.1	22	22.4
23	I was afraid I would not be able to communicate during my child’s treatment because of language barriers	22	14.8	13	25.5	9	9.2
***Perceptions of Mental Health Problems***							
8	I wanted to handle my child’s problems on my own	**110**	**73.8**	**42**	**82.4**	**68**	**69.4**
9	I felt embarrassed about my child’s problems	42	28.2	26	51.0	16	16.3
10	I felt embarrassed about needing help for my child’s problems	54	36.2	33	64.7	21	21.4
14	I did not think treatment could help with my child’s problems	56	37.6	28	54.9	28	28.6
19	I had difficulty motivating myself to seek treatment for my child	58	38.9	29	56.9	29	29.6
20	I felt like my child’s problems were normal for someone in their situation	**109**	**73.2**	**43**	**84.3**	**66**	**67.3**
***Perceptions of Mental Health Services***							
11	I was worried about being judged or criticized by my friends if I sought treatment for my child	52	34.9	27	52.9	**25**	**25.5**
12	I was worried about being judged or criticized by my family if I sought treatment for my child	**67**	**45.0**	**34**	**66.7**	**33**	**33.7**
13	I was afraid that my child would be committed to a hospital against my will	32	21.5	20	39.2	12	12.2
15	My child received treatment before, and it did not help with their problems	32	21.5	22	43.1	10	10.2
17	I was afraid treatment for my child would be too upsetting	55	36.9	**30**	**58.8**	**25**	**25.5**
18	I don’t trust mental health professionals	**53**	**35.6**	28	54.9	**25**	**25.5**
21	I was afraid my child would be treated badly in treatment because of their race or ethnicity	41	27.5	21	41.2	20	20.4
24	I was afraid of being deported for seeking treatment for my child	7	4.7	6	11.8	1	1.0
***Trauma-Related***							
25	I did not think that my child’s experiences were serious enough to warrant mental health treatment	**91**	**61.1**	33	64.7	**58**	**59.2**
26	I did not think that my child’s emotional/behavioral problems warranted mental health treatment.	**92**	**61.7**	31	60.8	**61**	**62.2**
27	I was afraid that we would not be believed or taken seriously by mental health professionals	46	30.9	24	47.1	22	22.4
28	I was afraid that information my child or I shared with mental health professionals would not be kept confidential	36	24.2	20	39.2	16	16.3
29	My child and/or I did not want to cause someone else to become upset	32	21.5	17	33.3	15	15.3
30	My child and/or I have a hard time trusting others	61	40.9	**34**	**66.7**	27	27.6
31	It would be hard for me and/or my child to let our guard down and let someone help us	78	52.3	**35**	**68.6**	43	43.9
***Cultural***							
32	I was afraid my parenting practices would be misunderstood and/or judged negatively	66	44.3	**35**	**68.6**	31	31.6
33	I thought I would be blamed for my child’s problems	66	44.3	33	64.7	33	33.7
34	In our culture, a mental health diagnosis would negatively affect my child’s future prospects	**76**	**51.0**	32	62.7	**44**	**44.9**
35	In our culture, it is considered a sign of weakness to seek mental health treatment	**84**	**56.4**	**35**	**68.6**	**49**	**50.0**

Note. Bolded values indicate the top two most frequently endorsed barrier within each subscale

#### Caregiver Support Questionnaire (CSSQ [38])

The CSSQ is a 24-item self-report measure examining caregiver perceived need for and utilization of a variety of youth mental health services in the past year. The CSSQ includes parallel questions assessing the perceived need for and use of commonly accessed formal mental health and informal support services among youth such as psychological counseling or therapy, medical doctors, mentorship programs, and school professionals. Participants rated each item as yes (1) or no (0) based on whether they perceived a need for or received that service for their child in the past year. Given our study’s focus on barriers to formal youth mental health services, we used only the 6 items reflecting perceived need for (3 items) and use of (3 items) psychological counseling or therapy, school-based mental health professionals, and family doctor/medical professional.

#### Traumatic Events Screening Inventory-Parent Report Revised (TESI-PRR; [39])

The TESI-PRR is a 24-item caregiver-reported questionnaire assessing lifetime occurrences of trauma exposure for children ages 3 to 17. Potentially traumatic events assessed include accidental trauma, domestic violence, community violence, physical and sexual abuse or assault. For the current study, one item about seeing/hearing about acts of war or terrorism was excluded. Total scores were calculated by summing endorsed events, with the potential range of 0–23. Studies have found a high correlation of the TESI-PRR with other measures of trauma exposure (*r* =.52 [40]) and adequate test-retest reliability between 0.50 and 0.79 [41].

#### Child & Adolescent Trauma Screen-Caregiver, 7–17 Years (CATS-C; [41])

The CATS-C questionnaire is a brief measure used to screen for posttraumatic stress disorder (PTSD). It assesses both lifetime history of potentially traumatic events and posttraumatic stress symptoms (PTSS) in children and adolescents between the ages of 7–17 years of age, with the caregiver as the informant. The CATS-C comprises 15 items measuring traumatic events, 20 items assessing DSM-5 PTSD symptoms, and 5 items measuring psychosocial functioning. Only the symptom portion of the scale was used for the current study. Total symptom scores ≥ 15 were considered clinically elevated based on the DSM-5 PTSD scoring criteria. Psychometric validation studies have shown excellent reliability, ranging between 0.88 and 0.94, and supported the underlying DSM-5 factor structure across different language versions [42]. In the current sample, internal reliability was excellent, α = 0.94.

#### Pediatric Symptom Checklist-17 (PSC-17 [43])

The PSC-17 is a shortened version of the original Pediatric Symptom Checklist (PSC [44]. It consists of 17 items completed by caregivers to assess psychosocial functioning and emotional and behavioral problems in children and adolescents aged 6 to 16 years. The PSC-17 assesses internalizing symptoms (e.g., depression and anxiety), externalizing symptoms (e.g., aggression and conduct problems), and attentional difficulties. Caregivers rated the frequency of each behavior in their child on a Likert scale from 0 = never to 2 = often. The total score is obtained by summing the scores across all items, with a clinical cutoff total score ≥ 15 (Range 0–34). Subscale scores for Attention, Externalizing, and Internalizing behaviors are calculated based on cutoffs for each, with scores ≥ 7 for Attention and Externalizing and ≥ 5 for Internalizing suggesting impairment in emotional or behavioral functioning [43, 45]. In the current sample, the internal reliability was good to acceptable for the total score (α = 0.87) and the three subscale scores: Internalizing (α = 0.69), Attention (α = 0.64), and Externalizing (α = 0.66).

### Data Quality

To ensure data quality, all responses were checked manually across multiple recommended indicators [46], including survey duration, responses on the two attention check items, inconsistent responses across items, and nonhuman responses to the open-ended barrier item. We excluded any responses that did not pass each of these checks. Of the 150 participants who completed the study via Prolific and passed these checks, one participant was removed from the final analytic sample due to reporting on a child younger than 6 years of age.

### Analysis

For the first aim, we first examined frequencies of endorsements for each barrier in the overall sample and by clinical status (i.e., within the clinical and non-clinical subgroups). If caregivers’ report of youth symptoms met the clinical threshold of at least one scale across the CATS-C (PTSD) and the PSC-17 (Internalizing, Externalizing, Attention Problems), they were in the clinical subgroup, and if they did not, they were part of the non-clinical subgroup. Responses to the barrier questions were dichotomized such that responses of 1 = *Not at all* was coded as no endorsement, whereas responses of 2 or greater were coded as endorsement. Next, we identified the two most frequently endorsed barriers in each barrier domain, again in the overall sample and by clinical status. We then examined patterns (i.e., proportion of the sample reporting need/use) of perceived need for and use of therapy, school-based mental health, and medical support services for youth mental health needs. Finally, we conducted exploratory correlational analyses to examine the bivariate associations between barriers and youth psychopathology, perceived need for services, and past-year service use, examining Pearson correlations between these variables and their associated *p*-values.

## Results

### Descriptive Analysis of Barriers

See Table 2 for frequencies and percentages of each barrier item.

#### Overall Sample

In the Structural/Practical subscale, the proportion of the sample endorsing each of the 10 barriers ranged from 14.8 to 63.1%. The most commonly endorsed items in the overall sample were (1) concerns about the cost of treatment (63.1%), and (2) not knowing where or from whom to seek treatment (60.4%). Insurance coverage and lack of time for treatment were also frequently endorsed, each by approximately half of sample (53%). Thus, the majority of caregivers in the sample noted concerns about cost and insurance coverage, time, and lack of knowledge about treatment resources as the key barriers in this domain. In the Perceptions of Mental Health Problems subscale, endorsements of the 6 items ranged from 28.2% to 73.8%. The most frequently endorsed items were (1) wanting to handle child’s problems on their own (73.8%), and (2) feeling like their child’s problems were normal (73.2%). Thus, self-reliance and low perceived need for treatment were the key barriers in this domain. The 8 items in the Perceptions of Mental Health Services subscale were endorsed relatively less frequently than items from the other scales, with endorsements ranging from 4.7% to 45.0%. Caregivers most frequently cited (1) worries about being judged/criticized by family members (45.0%), and (2) fears that treatment would be too upsetting for child (36.9%) as concerns. This suggested that concerns about stigmatizing responses from family members and the effect of treatment on child distress constituted the key barriers in this domain. Among the 7 trauma-related items, rates of endorsements ranged from 21.5% to 61.7%. Caregivers most frequently endorsed (1) belief that their child’s related emotional/behavioral problems were not serious enough to warrant treatment (61.7%), and (2) belief that their child’s experiences were not serious enough to warrant treatment (61.1%). Thus, the tendency to minimize potential impact of children’s traumatic experiences and related symptoms emerged as key barriers in this domain. Finally, rates of endorsements of the 4 cultural items ranged from 44.3% to 56.4%. The most commonly endorsed items reflected (1) culturally based stigma of mental health treatment (56.4%) and (2) worries that a mental health diagnosis would affect a child’s future prospects (51.1%), suggesting that caregivers were concerned about the culturally driven negative implications of seeking treatment for their children.

#### By Clinical Status

Based on caregiver report of youth psychopathology on the PSC-17 and the CATS-C, *n* = 51 (34.0%) of the youth had clinically significant levels of mental health concerns. Specifically, 29 (19.3%) youth were reported to have elevated internalizing problems, 13 (8.6%) had externalizing problems, 21 (14.0%) had attention difficulties, and 23 (15.3%) had elevated PTSD symptoms. Of note, we only designated youth as meeting PTSD symptom criteria if caregivers also endorsed a significant trauma history (i.e., one or more potentially traumatic events in their lifetime). Approximately 65% of the youth (*n* = 97) were reported by their caregivers as having experienced at least one traumatic event in their lifetime. The most frequently endorsed event was death of someone close (*n* = 47, 31.3% of the overall sample), followed by severe illness/injury of someone close (*n* = 29, 19.3%) and witnessing community violence (*n* = 24, 16.0%). Approximately 12% of youth (*n* = 18) had exposure to 4 or more potentially traumatic events in their lifetime.

The range of endorsements across all 35 barrier items was higher for the clinical subgroup (11.8% to 84.3%) compared to the nonclinical subgroup (1.0% to 69.4%). Patterns of the most frequently endorsed barriers within the clinical and non-clinical subsamples were similar to those observed in the overall sample, with a few differences within specific domains for the clinical subgroup and notable differences between the clinical and nonclinical subgroups. For the Structural/Practical subscale, in addition to cost, insurance coverage, and not knowing where to seek treatment, difficulty securing an appointment, not being able to choose the provider, dissatisfaction with available services, and concerns about racial ethnic match were each endorsed by 45%−59% of the clinical subgroup, a rate approximately twice that of the nonclinical subgroup. On the Perceptions of Mental Health Problems subscale, there were no differences between the two groups on the top two most frequently endorsed items. However, the other items from this scale, reflecting embarrassment about the child’s problems or needing help for child’s problems, doubts that treatment could help, and difficulty motivating oneself to seek treatment, were each endorsed by 51.0%−64.7% of the clinical subgroup, compared to 16.3%−29.6% of the nonclinical subgroup. On the Perceptions of Mental Health Services subscale, concerns that treatment would be too upsetting for the child was the second most frequently endorsed item in the clinical subgroup (58.8%), following worries about being judged/criticized by family members (66.7%). Among trauma-specific items, the statement “It would be hard for me and/or my child to let our guard down and let someone help us” was the most frequently endorsed (68.6%), in addition to difficulty trusting others (66.7%), belief that their children’s experiences were not serious enough to warrant treatment (64.7%), and belief that children’s emotional/behavioral problems did not warrant treatment (60.8%). Finally, among the cultural items, fears about being judged/misunderstood for their parenting style was most frequently endorsed (68.6%) along with the item reflecting culturally based stigma (68.6%). The remaining items, reflecting fear of being blamed for the child’s problems and mental health diagnosis negatively affecting a child’s future and were also highly endorsed (64.7% and 62.7%, respectively) in the clinical subgroup. Thus, the caregivers in the clinical subgroup cited more barriers overall and tended to endorse specific items within each subscale that were not endorsed at similar rates by caregivers in the nonclinical subgroup. See Table 2 for frequencies and percentages of endorsement of each barrier for each subgroup.

### Descriptive Analysis of Perceived Service Need and Past Year Service Use

Descriptive statistics concerning perceptions of service need and service use variables are presented in Table 3. Approximately half (51.0- 57.7%) of the caregivers in the overall sample endorsed perceived need for psychological counseling or therapy, school-based mental health, and medical services for their child in the past year. These rates were higher than the proportion of youth who met criteria for clinically elevated symptoms by caregiver report (34.0%). By contrast, only 20.1%, 34.9%, and 43.6% of caregivers endorsed past year use of therapy, school-based mental health, and medical professional services, respectively, for their children’s mental health needs. This disparity between perceived need and service use, and the greater use of medical and school-based services compared to psychological counseling or therapy, were also observed within the clinical and nonclinical subgroups. Despite the majority of caregivers in the clinical subgroup (74.5%) endorsing the need for counseling or therapy services for their children in the past year, only 18 of the 51 youth (35.3%) were reported to have received these services. Likewise, the proportion of caregivers in the clinical subgroup who reported using school-based services for their children was higher at 43.1%, but still smaller than the proportion of the caregivers who endorsed perceived need for school-based services (70.6%). Finally, the proportion of caregivers who reported using medical services was also smaller (51.0%) than the proportion of caregivers who endorsed this need (66.7%), though to a lesser extent when compared to other types of services.

**Table 3:** Descriptive statistics for service use variables in the overall sample and by clinical status

Variable	Overall sample (*N* = 149)			By Clinical Status			
				Clinical (*n* = 51)		Non-Clinical (*n* = 98)	
	*n*	*%*	Correlation with Barriers^1^	*n*	*%*	*n*	*%*
**Endorsement of Past-Year Perceived Need**							
Psychological counseling or therapy	75	50.3	*r =*.27, *p* <.001	38	74.5	37	37.8
A professional at school	86	57.7	*r =*.28, *p* <.001	36	70.6	50	51.0
A family doctor or other medical professional	76	51.0	*r =*.15, *p* =.061	34	66.7	42	42.9
**Endorsement of Past-Year Service Use**							
Psychological counseling or therapy	30	20.1	*r =*.13, *p* =.109	18	35.3	12	12.2
A professional at school	52	34.9	*r =*.20, *p* =.016	22	43.1	30	30.6
A family doctor or other medical professional	65	43.6	*r =*.16, *p* =.053	26	51.0	39	39.8

^1^Reflects Pearson correlations with mean global barrier scores

### Associations between Barriers, Youth Psychopathology, Perceived Service Need, and Service Use

In the overall sample, mean barrier scores were significantly and positively correlated with each of the symptom subscales, *r* =.44, 0.39, 0.44, and 0.47 for internalizing, attention, externalizing, and PSD symptoms, respectively, all *p*’s < 0.001 (see Table 4). These results indicated that the severity of caregiver-reported youth symptomatology was linked with greater endorsement of barriers to care. Likewise, mean barrier scores were positively correlated with perceived need for therapy, *r* =.27, *p* <.001, and school-based support services, *r* =.28, *p* <.001, but not with perceived need for medical support services, *r* =.15, *p* =.061; and positively correlated with past-year use of school-based support services, *r* =.20, *p* =.016, but not with therapy, *r* =.13, *p* =.109, or medical services, *r* =.16, *p* =.053 (see Table 3). Thus, the mean levels of barriers endorsed by caregivers were positively associated with the perceived need and past year use of specific types of support services, but not others.

**Table 4:** Pearson correlations between barriers and youth psychopathology variables (*N* = 149)

Variable	1	2	3	4	5	6	7	8	9	10
1. PSC Internalizing	-									
2. PSC Attention	0.436**	-								
3. PSC Externalizing	0.359**	0.604**	-							
4. CATS Trauma Symptoms	0.571**	0.512**	0.349**	-						
5. Barriers: Total	0.439**	0.390**	0.438**	0.472**	-					
6. Barriers: Trauma	0.244*	0.302**	0.282**	0.355**	0.811**	-				
7. Barriers: Cultural	0.361**	0.328**	0.388**	0.319**	0.807**	0.696**	-			
8. Barriers: Structural	0.455**	0.417**	0.417**	0.442**	0.829**	0.490**	0.530**	-		
9. Barriers: MH Problems	0.391**	0.285**	0.390**	0.433**	0.919**	0.674**	0.672**	0.680**	-	
10. Barriers: MH Services	0.398**	0.296**	0.395**	0.466**	0.888**	0.642**	0.657**	0.680**	0.919**	-
*M* (*SD*)	2.62 (2.00)	2.95 (2.71)	2.44 (2.60)	6.85 (9.25)	1.84 (0.66)	1.87 (0.74)	2.03 (1.10)	1.85 (0.77)	1.81 (0.74)	1.54 (0.70)
Range	0–8	0–10	0–14	0–40	1.00–3.6	1.00–4.14	1.00–5.00	1.00–4.10	1.00–3.92	1.00–3.62

*p <.01, **p <.001

PSC = Pediatric Symptom Checklist; CATS = Child and Adolescent Trauma Screen; MH = Mental Health

## Discussion

The aims of the current study were to (1) describe key barriers to youth mental health services as reported by AsAm caregivers, (2) describe patterns of service use, and (3) explore links between barriers with youth mental health symptoms and mental health service use. We included previously validated barrier domains, namely, structural, perceptions of mental health problems, and perceptions of mental health services. Drawing from prior qualitative research [36, 37], we additionally included trauma-specific and cultural barriers. Given the lack of prior quantitative research in this area, we did not include any hypotheses regarding key barriers. Likewise, correlational analyses were conducted on an exploratory basis.

In our sample of AsAm caregivers, endorsements of structural, perceptual, cultural, and trauma-specific barriers ranged from 4.7% to 73.8%, aligning with existing literature on other racial-ethnic minority populations showing elevated barriers to care [33, 47]– [48]. Our results suggested that AsAm caregivers overall perceive significant and multifaceted barriers when considering mental health services for their children. Key barriers included concerns about cost and lack of access to information about available treatment resources in the structural domain, preference for self-reliance and low perceived need for services in the perceptions of mental health problems domain, and concerns about being judged or criticized by family members and fears that treatment would elicit child distress in the perceptions of mental health services domain. Among trauma-specific barriers, key barriers included beliefs that children’s (potentially traumatic) experiences or resulting emotional/behavioral problems were not serious enough for treatment. Among cultural barrier items, being seen as “weak” for seeking treatment and concerns about the effects of mental health diagnoses on children’s futures emerged as key barriers. These findings highlight the importance of addressing both universal barriers and those uniquely relevant to the AsAm community. Specifically, culturally based stigma and historical traumas in the AsAm community are likely to shape help seeking behavior across generations, affecting youth access to mental health supports. Without identifying ways to address these salient factors, efforts to address universal barriers (e.g., financial) may not fully resolve the treatment disparities for AsAm youth.

Consistent with prior studies conducted outside the U.S [49]., endorsements of barriers were higher for caregivers who perceived their children to have clinically significant levels of mental health symptoms across domains, relative to those who did not report clinically significant levels of symptoms for their children. We note here the possibility that some youth who were deemed to not experience clinical levels of symptoms may, in fact, have been symptomatic if they were asked to also self-report their symptoms, and vice versa. Nonetheless, caregivers who perceived their youth to have significant levels of mental health symptoms also perceived more barriers to accessing care for these children. This finding was also supported by positive and significant correlations between mean barrier scores and each of the youth mental health symptom scores. This is a concerning pattern, potentially suggesting that the caregivers whose youth have the *most apparent need* for services also have the *most difficulty* accessing them, warranting further study. Additionally, key barriers for the clinical subgroup diverged at times from the barriers in the nonclinical subgroup; for instance, concerns reflecting difficulty trusting others, embarrassment for needing help for children’s problems, fear of being judged/misunderstood for their parenting style, and being blamed for their children’s problem were frequently endorsed by the caregivers in the clinical subgroup only. Further study of these unique barriers, which appear to highlight caregivers’ desire to avoid shame, may be key to addressing the low rates of service use among AsAm youth in need of mental health care.

Service use data in the current sample revealed that caregivers sought support most frequently from medical professionals, followed by school-based mental health professionals, and were least likely to have used psychological counseling/therapy for their children’s mental health needs. This was despite approximately half of the caregivers in the sample endorsing a perceived need for each of these services for their children in the past year. The low rate of counseling/therapy service use was also true in the clinical subgroup, and more youth in this group were reported as having accessed both medical and school-based supports compared to counseling/therapy in the past year. The pattern of service utilization could be due to Asian Americans placing a greater emphasis on identification and treatment of somatic symptoms rather than emotional symptoms [50], and the ability of school-based mental health care to address many structural barriers to care. This apparent acceptability of medical services and school-based supports for youth mental health is promising given the persistent treatment gaps in the AsAm community. Simultaneously, for many common youth mental health conditions (e.g., anxiety, PTSD), evidence-based therapies typically delivered in outpatient mental health settings remain first-line interventions. As such, task-shifting the delivery of evidence-based therapies in settings reported as more acceptable for Asian Americans, such as with teachers, social workers, and primary care physicians, may be one way to improve the uptake and engagement with such interventions. It is also crucial that researchers, practitioners, funders, and policymakers attend to the pressing need to improve the delivery, acceptability (i.e., fit, relevance) and tolerability (i.e., feasibility of sustained participation) of well-established evidence-based therapies for AsAm youth and families (e.g., CBT [51]).

Exploratory correlational analyses revealed positive associations between barriers and perceived need for counseling/therapy and school-based support, but not medical services, and between barriers and past-year use of medical services but not therapy or school-based supports. It is likely that these correlations at least partly reflect common method variance given our sole reliance on caregiver reporting of these constructs. At the same time, it is possible that greater endorsement of barriers in specific domains could result in greater use of specific services that effectively mitigate those barriers (e.g., structural barriers for school-based supports). We also did not ask about the characteristics of services received, including dosage or effectiveness, limiting our interpretation of these data. Future research should assess these characteristics of services to clarify the links between barriers and services sought and/or received and employ a prospective design to more accurately estimate the directionality of these effects. Because of known barriers to research participation among Asian Americans, future work in this area will also require community engagement to build trust and collaboration directly with community members.

### Study Strengths & Limitations

The current study also has notable strengths, including the inclusion of AsAm caregivers representing diverse ethnic backgrounds and socioeconomic status. This is the first study, to our knowledge, that has attempted to quantitatively characterize the barriers to mental health services for AsAm youth and families, who are consistently the least likely racial group to use mental health services in the U.S. Our findings contribute to a body of ongoing work attempting to elucidate barriers and patterns of service use by AsAm caregivers for youth mental health needs, who remain understudied despite persistent disparities in service use. We also note several important limitations of our study. First, we used a cross-sectional design, which could potentially overlook symptom fluctuations and changes in caregiver perceptions over time. This design also precluded the ability to test whether youth mental health symptoms increase barriers over time, and whether barriers predict later service use. Addressing these limitations will be an important next step in this research. Second, symptoms and trauma histories were reported by caregivers only. Caregivers’ reports are an important source of information, but information from their perspectives may not correspond with the full range of children’s experiences. Nevertheless, caregivers are the gatekeepers for youth mental health services, and their perception of barriers has critical implications for youth access to care. Relatedly, given the under recognition of and heightened sensitivity to mental health problems within the AsAm community, caregivers could have been susceptible to social desirability bias, underreporting youth symptomology and trauma histories. Future studies may benefit from using a longitudinal design and incorporating youth self-report, in addition to caregivers’ perspectives.

We note that the generalizability of our findings is limited by our use of Prolific for recruitment. Participants on Prolific may have differed from the larger population of AsAm caregivers in notable ways, such as in their English language fluency, levels of acculturation, socioeconomic status, and other factors potentially affecting their perceptions and experiences of mental health services for their children. For instance, only 22 caregivers in our sample (14.8%) reported concerns about language barriers, which we suspect is not reflective of the broader AsAm community. We again note the documented and significant barriers to research participation for the AsAm community, and specifically call attention to the need for more funding and resources to address the diverse language needs in research participation to ensure more representative data. Additionally, given the relatively modest sample size, we were not able to disaggregate our data to examine important subgroup differences (e.g., by ethnicity or immigration status). We acknowledge the rich diversity within the AsAm community, and such, note that findings here may not elucidate the specific barriers experienced by AsAm subgroups with unique cultural differences.

## Conclusion

These limitations notwithstanding, our findings suggest that AsAm caregivers contend with multifaceted barriers when considering support services for their children’s mental health needs. Findings also highlight disparities in the use of services for the subset of youth reported to have clinically significant mental health symptoms, with only about a third of these youth accessing therapy or counseling services in the past year. These results suggest that AsAm families experience both universal structural barriers (e.g., cost, insurance coverage), as well as culturally specific barriers (e.g., emphasis on self-reliance, misalignment between cultural values and services) that uniquely shape help-seeking behaviors. Future research should examine the interplay of these factors and their effects on youth mental health service use, with the goal of ensuring that AsAm youth are able to access and engage with evidence-based mental health services.

## Data Availability

The data for this study are available from the corresponding author by request.
